# Experimental Study of the Influence of Ink Properties and Process Parameters on Ejection Volume in Electrohydrodynamic Jet Printing

**DOI:** 10.3390/mi9100522

**Published:** 2018-10-16

**Authors:** Lei Guo, Yongqing Duan, YongAn Huang, Zhouping Yin

**Affiliations:** State Key Laboratory of Digital Manufacturing Equipment and Technology, Huazhong University of Science and Technology, Wuhan 430074, China; hustgl@hust.edu.cn (L.G.); duanyongqing@hust.edu.cn (Y.D.)

**Keywords:** electrohydrodynamic jet printing, viscosity, conductivity, applied voltage, nozzle-to-substrate distance, regression model, ejection volume

## Abstract

Electrohydrodynamic jet (e-jet) printing has very promising applications due to its high printing resolution and material compatibility. It is necessary to know how to choose the printing parameters to get the right ejection volume. The previous scaling law of the ejection volume in e-jet printing borrows the scaling law of the ejection volume of an unstable isolated droplet charged to the Rayleigh limit. The influence of viscosity, applied voltage amplitude, and nozzle-to-substrate distance on the ejection volume in e-jet printing was not taken into account in the scaling law. This study investigated the influence of viscosity, conductivity, applied voltage, and nozzle-to-substrate distance on the ejection volume. The ejection volume increases with viscosity and decreases with applied voltage and nozzle-to-substrate distance. The average electric field was kept unchanged while changing the nozzle-to-substrate distance by changing the applied voltage according to the electric field model of a semi-infinite wire perpendicular to an infinite large planar counter electrode. The ejection volume decreases with conductivity as $V\sim K^{-0.6}$, which is different from the previous scaling law, which concludes that $V\sim K^{-1}$. Finally, a model about the relation between the ejection volume and four parameters was established by regression analysis using a third-order polynomial. Two more experiments were done, and the predicted results of the fitted model accorded well with the experiments. The model can be used to choose the ink properties and process parameters to get the right ejection volume.

## 1. Introduction

Recently, electrohydrodynamic jet (e-jet) printing has attracted much attention due to its high printing resolution and material compatibility [1,2,3,4]. It can generate small droplets (diameter < 1 µm) and it is adaptable to liquids with a wide range of viscosity (1–1000 mPa·s) [5,6,7,8]. It has been used to fabricate transparent electrodes [9], thin film transistors [2], DNA microchips [10], quantum dots for light-emitting diodes (LEDs) [11], 3D structures [12,13], and others [14,15,16]. During e-jet printing, the pendant drop at the apex of the nozzle can be deformed into a conical meniscus (Taylor cone) under a high electric field. When the electrostatic force overcomes the surface tension of the pendant drop, a thin slender jet will be emitted from the apex of the Taylor cone, and then the jet ejects liquid on the substrate [17,18,19]. By adjusting the physical properties of liquids (such as surface tension, viscosity, density, and conductivity) or the process parameters (such as applied voltage, nozzle-to-substrate distance, and flow rate), several pulsation modes can be observed in e-jet printing, such as stable cone-jet mode, micro-dripping mode [20], and unstable cone-jet mode [21].The influence of various parameters on the stable cone-jet mode [22] and the micro-dripping mode [23] have been discussed. The unstable cone-jet is most commonly used due to its wide process window. However, the influence of various parameters on the unstable cone-jet is still not clear, and the precise control of droplet volume is still challenging.

Fernandez [24] theoretically analyzed the ejected liquid volume of an isolated unstable droplet electrified to the Rayleigh limit. Chen et al. [25] adopted the results of Fernandez to analyze the ejection volume and pulsation frequency in e-jet printing. In the scaling law, the influence of viscosity on the ejection volume was not taken into account. In e-jet printing, the applied voltage and nozzle-to-substrate distance are important parameters that influence the ejection volume but are not related to the explosion of an electrified droplet, so their influence on the ejection volume also needs to be investigated. Yuan et al. [26,27] and Xu et al. [17] analyzed the influence of voltage pulsation frequency and supplied flow rate on the ejection volume when a pulsed voltage was applied. Syringe pumps were used in their experiments. Rahman et al. [28] analyzed the influence of applied pressure, the duty ratio, and applied voltage amplitude on the feature size in pulsed electrohydrodynamic jet printing. Since it is hard to get a scaling law describing the effects of various parameters on the ejection volume theoretically, some people have used statistical methods to predict and optimize the printing results. Ball et al. [29] established a relationship between several parameters (applied voltage, supplied flow rate, and stand-off height) and printed droplet diameter through regression analysis of the experimental data. Das et al. [30] optimized the applied pressure, stand-off height, and applied voltage to achieve a smaller droplet diameter and higher printing frequency by combining weighted principal component analysis (WPCA) and the Taguchi method. Park et al. [31] also predicted the droplet diameter and line width through experiments in pulsed electrohydrodynamic jet printing.

In this paper, the influence of viscosity, conductivity, applied voltage, and nozzle-to-substrate distance on the ejection volume was analyzed through systematic experiments under DC applied voltage. The average electric field strength on the surface of the meniscus was kept unchanged while changing the nozzle-to-substrate distance by changing the applied voltage according to the electric field model of a semi-infinite wire perpendicular to an infinite large planar counter electrode. The mechanisms underlying the printing results were determined. Regression analysis was used to get a fitted model defining the relationship of various parameters (viscosity, conductivity, applied voltage, and nozzle-to-substrate distance) and ejection volume from experimental data. The central component design (CCD) method was used to design the experiments. Analysis of variance (ANOVA) showed the significance of the regression model. Two more experiments were done, and the prediction value of the model accorded well with the experimental results. The model can be used to choose the ink properties and the process parameters to get the right ejection volume.

## 2. Materials and Methods

**Figure 1:**
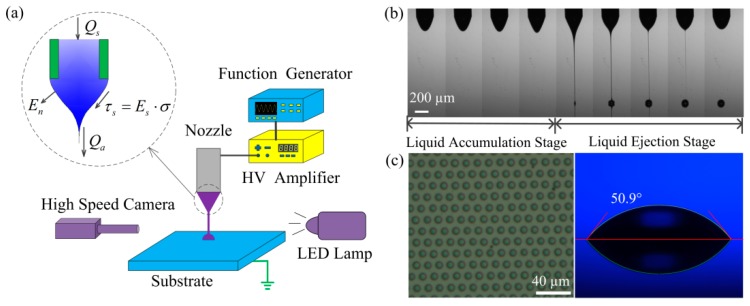
The schematic of e-jet printing: (**a**) the schematic of the e-jet printing setup; (**b**) the ejection process of e-jet printing; (**c**) the printed dot array on the substrate, where the right image represents the contact angle of the droplet with the substrate.

Figure 1a is the schematic of the experimental setup. A metallic nozzle (DPN-30G-1, Musashi Engineering, Inc., Tokyo, Japan) was connected to a syringe. The inner diameter of the nozzle is 160 μm and the outer diameter is 260 μm. The diameter of the DPN-32G-1 nozzle is smaller, but the printing frequency will be very slow due to its small inner diameter (110 μm). So, we chose the DPN-30G-1 nozzle. The apex of the nozzle is flat. All the experiments were done by the same nozzle in order to reduce the uncertainties in experiments. The syringe was mounted on an automatic Z-axis motion stage which can be used to adjust the nozzle-to-substrate distance precisely. The upper end of the syringe was connected to an air supply. The applied voltage was generated by a waveform generator (33500B Series, Keysight Technologies, Osaka, Japan) and amplified 1000 times by a high-voltage amplifier (MODEL 609E-6, Trek Inc, Lockport, NY, USA). The high-voltage side was connected to the metallic nozzle and the zero-voltage side was connected to the substrate holder, which was made of a copper plate. The jetting process was visualized by a high-speed camera (Dimax HD, PCO AG, Kelheim, Germany) with a zoom lens (magnification 1.16~13.92, Navitar Inc., Rochester, NY, USA) and a high-power LED lamp (PLED-100, Ti-times Inc, Shenzhen, China).

The liquids were various mixtures of glycerine, deionized water, and sodium chloride (NaCl) aqueous solutions. The viscosities of the liquids were adjusted by changing the volume ratio of glycerine to NaCl solution + deionized water. By changing the volume ratio of NaCl solution to deionized water, the conductivity of the liquid can be adjusted. The real compositions and properties of various liquids are listed in Table A1. The viscosities, conductivities, surface tensions, and contact angles were measured by a viscometer (DHR-1, TA Instruments, New Castle, UK), a conductivity meter (DDSJ-308A, INESA Scientific Instrument, Shanghai, China), a surface tension meter (QBZY-1, FangRui Instrument, Shanghai, China), and a contact angle meter (SL200B, Kino Industry CO, Ltd, Somerville, MA, USA), respectively. The minimum (maximum) surface tension is 64.1 mN/m (65.7 mN/m) for liquid with viscosity of 100 mPa·s (20 mPa·s). The minimum (maximum) density is 1172.2 $\mathrm{kg}/m^{3}$ (1219.6 $\mathrm{kg}/m^{3}$) for liquid with viscosity of 20 mPa·s (100 mPa·s). The minimum (maximum) relative dielectric constant is 53.2 (58) for liquid with viscosity of 100 mPa·s (20 mPa·s). Although the composition changed for different liquids, the densities, the surface tensions, and the relative dielectric constants changed only a little and can be treated as constant in the analysis. The density was approximated by the average value of the maximum density and the minimum density, which is 1195.9 $\mathrm{kg}/m^{3}$. The surface tension was approximated by the average value of the maximum surface tension and the minimum surface tension, which is 64.9 mN/m.

In the experiments, the Taylor cone ejected droplets intermittently in a specific frequency under a constant DC voltage. Figure 1b is the ejection process, taken by the high-speed camera. As shown in Figure 1b, there are two stages in one circulation in an unstable cone-jet: the liquid accumulation stage and the liquid ejection stage. The meniscus wet the outer diameter for all the experiments. The experiments were done in a clean room at room temperature (20 °C). The substrate stage moved laterally at a constant speed of 20 mm/s during printing. The dots were printed on a silicon wafer. All the experiments were done on the same silicon wafer in order to guarantee that the contact angles for all the printed dots were the same. The silicon wafer was cleaned by deionized water after each experiment. Then, the silicon wafer was dried by hot air for 2 min in order to remove the residual water film on the silicon wafer, which will otherwise influence the contact angle of the printed dots. The left picture of Figure 1c was taken by a microscope (DSX-510, Olympus Corporation, Tokyo, Japan) and the right picture of Figure 1c was taken by the camera on the contact angle meter. As shown in Figure 1c, the dots printed on the substrate have regular shapes. The diameters of the droplets and the distances between adjacent droplets can be directly measured. The contact angle of the droplet on the substrate is 50.9°. The volume of the droplets can be calculated as the volume of a spherical cap.

## 3. The Influence of Viscosity, Conductivity, Applied Voltage, and Nozzle-to-Substrate Distance on Ejection Volume

### 3.1. Influence of Liquid Viscosity on Ejection Volume

**Figure 2:**
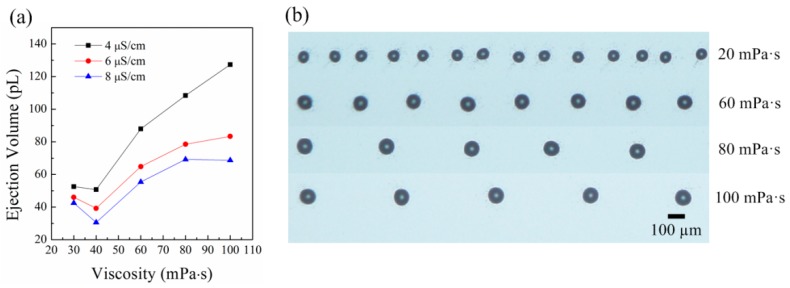
The ejection volumes for liquids with different viscosities: (**a**) the relation of ejection volume and viscosity; (**b**) the printed droplets on the substrate for liquids with different viscosities. The applied voltage was 2150 V. The nozzle-to-substrate distance was 1.5 mm. The moving speed of the substrate was 20 mm/s.

Figure 2 shows the influence of viscosity on the ejection. Figure 2a shows that the ejection volume increases as the viscosity increases. When the viscosity of the liquid is small, the ejection volume decreases slightly with viscosity. By dimensional analysis, the relation of ejection volume (V) and other parameters can be expressed as [32]

(1) $$\frac{V}{V_{c}}=F_{1}(\varepsilon_{r},\frac{t_{e}}{t_{c}},\frac{\mu }{\sqrt{\rho \gamma d_{n}}},\frac{H}{d_{n}},\frac{U}{\ln(8H/d_{n})\sqrt{\gamma d_{n}/\varepsilon_{0}}})$$

There are five dimensionless parameters that influence the ejection volume. It is known that the jet diameter ($d_{j}$) at the beginning of ejection is [33]

(2) $$d_{j}\sim {\left(\mu^{3}\varepsilon_{r}\varepsilon_{0}/(\rho^{2}\gamma \sigma )\right)}^{\frac{1}{3}}$$

From Equation (2), one can see that as μ→0, with the other parameters unchanged, $d_{j}\to 0$. The electric shear stress cannot accelerate the liquid in the cone through a viscous force as μ→0. That means jet ejection does not happen, so the ejection volume V→0. In summary, V→0 as $\mu /\sqrt{\rho \gamma d_{n}}\to 0$. From the theory of dimensional analysis, the influence of the parameter $\mu /\sqrt{\rho \gamma d_{n}}$ on the ejection volume cannot be ignored no matter how small the viscosity of the liquid is. Figure 2b shows the printed droplets on the silicon wafer for liquids with different viscosities. The cone ejected liquid at a certain frequency under a constant applied voltage. The substrate moved at a constant speed of 20 mm/s simultaneously. Because the duration of jet ejection was short, the ejected liquid deposited at the same position on the silicon wafer and formed a droplet. A series of droplets with equal distance formed on the silicon wafer as the substrate moved. It can be seen that the diameter of the droplet increases with viscosity. So, the resolution will decrease if the viscosity of the liquid increases. The distance between adjacent droplets also increases with viscosity. That means the pulsation frequency decreases as the viscosity of the liquid increases. The decrease of the pulsation frequency is caused by the decrease of the supplied flow rate with viscosity and the increase of the ejection volume.

### 3.2. Influence of Liquid Conductivity on Ejection Volume

**Figure 3:**
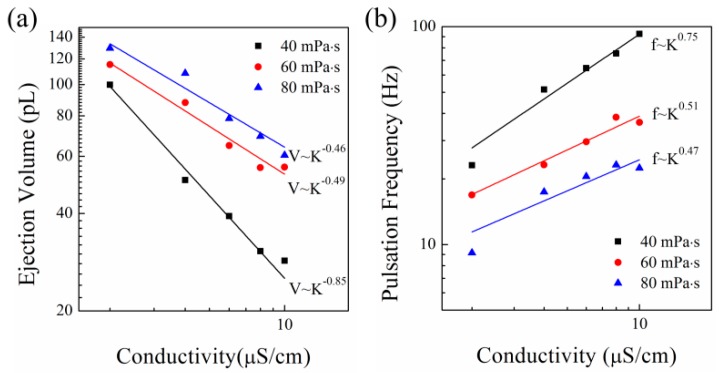
The ejection volume and pulsation frequency for liquids with different conductivities: (**a**) the relation of ejection volume and conductivity; (**b**) the relation of pulsation frequency and conductivity. The applied voltage was 2150 V. The nozzle-to-substrate distance was 1.5 mm.

Figure 3 shows the influence of conductivity on the ejection. Figure 3a shows the relation of the ejection volume and conductivity. The decrease rate of the liquids with conductivity for liquids with viscosity of 20 mPa·s is much larger than those for liquids with viscosity of 40 mPa·s and 60 mPa·s. By averaging the three exponents −0.46, −0.49, and −0.85, it can be derived that the ejection volume decreases with conductivity as $V\sim K^{-0.6}$. One can get smaller dots by increasing the conductivity of the liquid. The experimental results are different from the previous theoretical analysis [25], which concluded that the relation of the ejection volume and the conductivity is $V\sim K^{-1}$. Figure 3b shows the relation of the pulsation frequency and conductivity. By averaging the three exponents 0.47, 0.51, and 0.75, it can be derived that the pulsation frequency increases with conductivity as $f\sim K^{0.58}$. So, the increase rate of the pulsation frequency is nearly the same as the decrease rate of the ejection volume. It is known that the equivalent supplied flow rate in e-jet printing can be expressed as [25,34]

(3) $$Q_{s}\sim \frac{\pi d_{n}^{4}}{128\mu L_{n}}(\frac{\varepsilon_{0}E_{n}^{2}}{2}-\frac{4\gamma }{d_{n}})\;$$

$E_{n}$ depends on the applied voltage but is not influenced by the conductivity of the liquid. In Equation (3), when the electric normal stress ($\varepsilon_{0}E_{n}^{2}/2$) overcomes the surface tension stress ($4\gamma /d_{n}$), the liquid in the nozzle is pulled out. Although the electric shear stress is different for liquids with different conductivities, it is small compared to the electric normal stress, so its influence on the supplied flow rate can be neglected. From Equation (3), it can be derived that the supplied flow rate does not change with the conductivity of the liquid. The relation of supplied flow rate, ejection volume, and pulsation frequency can be expressed as

(4) $$Q_{s}=V\cdot f\;$$

Because the supplied flow rate does not change with conductivity, the decrease rate of the ejection volume is equal to the increase rate of pulsation frequency. So, if only the conductivity of the liquid is changed, the change of the ejection volume can be known by the change of the pulsation frequency.

Although the electric shear stress does not influence the supplied flow rate, it has a significant influence on the ejection volume been pulled out [33]. The electric shear stress ($\tau_{s}$) can be expressed as

(5) $$\tau_{s}=E_{s}\cdot \sigma \;$$

In the bulk of the Taylor cone, the velocity of the liquid is slow. The surface charge density is approximately the same with the electrostatic limit, which can be expressed as

(6) $$\sigma \sim \sigma_{e}\;$$

where $\sigma_{e}$ is the equilibrium surface charge density, which makes the cone equipotential and is not related to conductivity. Suppose that the average cone angles (θ) are the same for liquids with different conductivities, then the tangential electric field at the cone surface is [35]

(7) $$E_{s}=\frac{I}{2\pi (1-\cos\theta )r_{c}^{2}K}\;$$

The current can be expressed as $I=f(\varepsilon_{r}){\left(\frac{\gamma KQ_{e}}{\varepsilon_{r}}\right)}^{\frac{1}{2}}$. Equations (5)–(7) lead to

(8) $$\tau_{s}=\frac{f(\varepsilon_{r})}{2\pi (1-\cos\theta )r_{c}^{2}}{\left(\frac{\gamma Q_{e}}{\varepsilon_{r}K}\right)}^{\frac{1}{2}}\sigma_{e}\;$$

It can be seen that the electric shear stress decreases with conductivity. So, larger conductivity corresponds to less liquid been accelerated by the shear stress and smaller volume been ejected out. From Equation (2), it is clear that the emitted jet diameter ($d_{j}$) at the beginning of ejection decreases as the conductivity of the liquid increases. As K→∞, with the other parameters unchanged, $d_{j}\to 0$. That means jet ejection will not happen, so the ejection volume V→0. From Equation (1), when K→∞ and the other parameters are constant, $t_{e}/t_{c}\to 0$. In summary, V→0 as $t_{e}/t_{c}\to 0$. So, $t_{e}/t_{c}$ is a parameter that influences the ejection volume and cannot be ignored no matter how small it is. Since conductivity influences the ejection volume and the pulsation frequency, the pulsating phenomenon cannot be treated as a mechanical oscillation phenomenon which ignores the volume loss during jet ejection.

**Figure 4:**
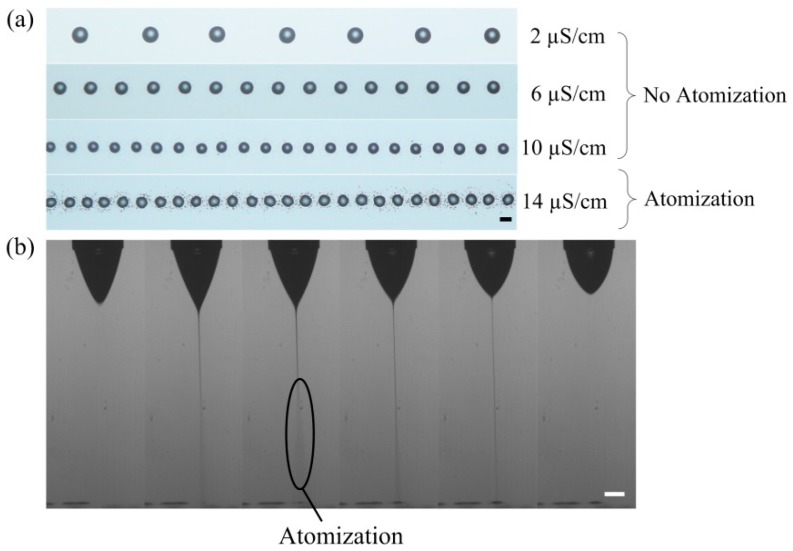
The atomization in e-jet printing: (**a**) the image of printed droplets on a silicon wafer; (**b**) the jetting process for liquid with a conductivity of 14 μS/cm. The time interval between adjacent images was 143 μs. The viscosity of the liquids was 60 mPa·s. The applied voltage was 2150 V. The nozzle-to-substrate distance was 1.5 mm. The moving speed of the substrate was 20 mm/s. Scale bar represents 100 μm.

Figure 4 shows the image of printed droplets of liquids with different conductivities. As shown in Figure 4a, as the conductivity of the liquid increases, both the average spacing between adjacent droplets and the diameters of the droplets decrease. Smaller average spacing between adjacent droplets corresponds to higher pulsation frequency. Smaller droplet diameter corresponds to a smaller volume ejected during each pulsation. When the conductivity of the liquid is smaller than 10 μS/cm, there are no small satellite droplets around the main drop. However, for the liquid with a conductivity of 14 μS/cm, there are many small droplets around the main drop. Figure 4b shows the jetting process of liquid with a conductivity of 14 μS/cm. As the conductivity of the liquid increases, the average diameter of the jet decreases and the density of the surface charge on the jet is higher. When the conductivity of the liquid is 14 μS/cm, the jet will atomize before reaching the substrate, which results in the existence of tiny drops around the main drop. The degree of atomization is small at the beginning and the end of the jet ejection, and the degree of atomization is large at the middle of the jet ejection. So, the atomization highly depends on the formulation of the liquid.

### 3.3. Influence of Applied Voltage on Ejection Volume

**Figure 5:**
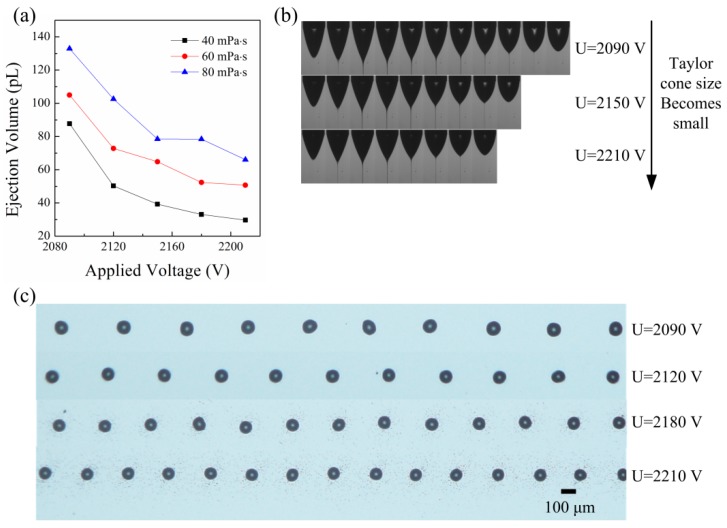
The ejection volume for different applied voltages: (**a**) the relation of the ejection volume with the applied voltage; (**b**) the images of the ejection process for different applied voltages; (**c**) the printed droplets on the silicon wafer for different applied voltages. The viscosity of the liquid for (**b**) and (**c**) was 60 mPa·s. The conductivity of the liquids was 6 μS/cm. The nozzle-to-substrate distance was 1.5 mm. The moving speed of the substrate was 20 mm/s.

Figure 5 shows the influence of the applied voltage on the ejection volume. Since the electric stress should overcome the surface tension stress, the applied voltage can change only in a small range. If the applied voltage is too small, jet ejection will not happen. If the applied voltage is too large, air breakdown happens. However, the applied voltage still has a big influence on the ejection process. Figure 5a shows that the droplet volume decreases with the applied voltage. Figure 5b shows the ejection processes for different applied voltages. It can be seen that the Taylor cone becomes small and the ejection duration becomes short as the applied voltage increases. The size of the Taylor cone has a big influence on the ejection volume. The small Taylor cone results in a small ejection volume. Figure 5c shows the printed droplets on the substrate for different applied voltages. As the applied voltage increases, the diameter of the droplet becomes small and the distance between adjacent droplets decreases. That means the pulsation frequency increases with the applied voltage. As the applied voltage increases, the normal electric field on the Taylor cone surface becomes large. From Equation (3), the supplied flow rate will increase with the normal electric field on the Taylor cone surface. So, from Equation (4), the increase of the pulsation frequency is partly caused by the decrease of the ejection volume and partly caused by the increase of the supplied flow rate.

### 3.4. Influence of Nozzle-to-Substrate Distance on Ejection Volume (the Average Electric Field on the Meniscus Kept Unchanged)

Since the real applied voltage must change with the nozzle-to-substrate distance, it is beneficial to find a dimensionless voltage, which should not change when the nozzle-to-substrate distance changes. The electric field at the tip of the nozzle ($E_{n}$) can be expressed by the electric field model of a semi-infinite wire perpendicular to an infinite large planar counter electrode as [34]

(9) $$E_{n}=4U/d_{n}\ln(8H/d_{n})\;$$

Since the electric stress ($\varepsilon_{0}E_{n}^{2}/2$) should overcome surface tension stress ($4\gamma /d_{n}$) in e-jet printing, it can be expressed as

(10) $$\frac{1}{2}\varepsilon_{0}E_{n}^{2}\sim \frac{4\gamma }{d_{n}}\;$$

Combined with Equations (9) and (10), the dimensionless applied voltage ($U{}^{\prime }$) can be expressed as

(11) $$U{}^{{}^{\prime }}\sim U/(\ln(8H/d_{n})\sqrt{\gamma d_{n}/\varepsilon_{0}})\;$$

So, the average electric field on the meniscus will be kept unchanged by keeping $U{}^{\prime }$ unchanged.

**Figure 6:**
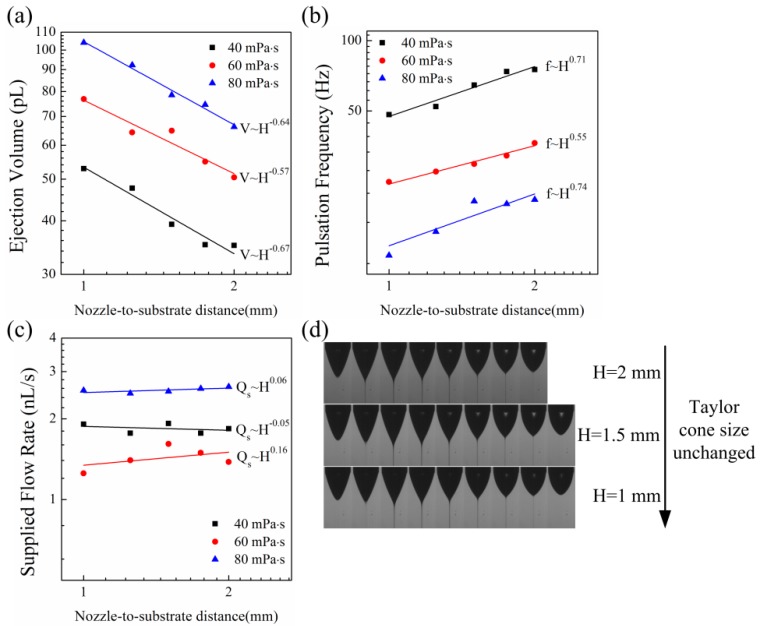
The ejection for different nozzle-to-substrate distances: (**a**) the relation of the ejection volume and nozzle-to-substrate distance; (**b**) the relation of the pulsation frequency and nozzle-to-substrate distance; (**c**) the relation of the supplied flow rate and nozzle-to-substrate distance; (**d**) the image of the ejection process for different nozzle-to-substrate distances. The conductivity of the liquid was 6 μS/cm. The dimensionless applied voltage was $U{}^{\prime }=9.424$.

Figure 6a shows the relation of the ejection volume and nozzle-to-substrate distance. By averaging the three exponents −0.64, −0.57, and −0.67, it can be derived that the ejection volume decreases with the nozzle-to-substrate distance as $V\sim H^{-0.63}$. The biggest nozzle-to-substrate distance was 2 mm in the experiments. If the nozzle-to-substrate distance is larger than that, the printed droplets become atomized and it will be difficult to calculate the ejected liquid volume. Although the electric field strength remains unchanged while changing the nozzle-to-substrate distance, the distribution of the normal electric field on the meniscus surface changes for different nozzle-to-substrate distances. Since the meniscus is equipotential, the bottom of the meniscus is nearer to the substrate, so the electric field is higher than the electric field of other places of the meniscus. This will make the meniscus eject more liquid on the substrate. When the nozzle-to-substrate distance is small, this effect is significant. However, as the nozzle-to-substrate distance increases, the size of the meniscus becomes small compared to the nozzle-to-substrate distance. The difference of the electric field strength on different places of the meniscus becomes small, so the ejection volume decreases. Figure 6b shows the relation of the pulsation frequency with nozzle-to-substrate distance. By averaging the three exponents 0.71, 0.55, and 0.74, it can be derived that the pulsation frequency increases with the nozzle-to-substrate distance as $f\sim H^{0.67}$. In order to demonstrate that the average electric field strength on the meniscus does not change with the nozzle-to-substrate distance, the supplied flow rate is presented in Figure 6c. By averaging the three exponents 0.06, −0.05, and 0.16, it can be derived that the relation of the supplied flow rate and the nozzle-to-substrate distance is $Q_{s}\sim H^{0.06}$. So, the supplied flow rate does not change with the nozzle-to-substrate distance. According the Equation (3), this demonstrates that the average normal electric field on the meniscus is unchanged. From Equation (4), the increase rate of the pulsation frequency with the nozzle-to-substrate distance is equal to the decrease rate of the ejection volume with the nozzle-to-substrate distance. So, the change of the ejection volume can be known by the change of the pulsation frequency. Figure 6d shows the images of the ejection processes for different nozzle-to-substrate distances. From the previous section, it is known that the size of the Taylor cone changes with the normal electric field strength on the meniscus. By keeping the dimensionless voltage unchanged while changing the nozzle-to-substrate distance, the influence of the size of the Taylor cones is minimized.

## 4. Regression Model

Since it is hard to produce an analytical model of the relation between the ejection volume and the various parameters, it is useful to get the relationship between the ejection volume and the various parameters by regression analysis of the experimental data. The parameters were non-dimensionalized in the regression analysis. As the dielectric constants of the liquids were nearly the same in the experiments, only the other four parameters were considered in the regression analysis. The ejection volume was non-dimensionalized as $V{}^{\prime }=V/d_{n}^{3}$. The conductivity of the liquid was non-dimensionalized as $K{}^{\prime }=K/\varepsilon_{0}\cdot {\left(\rho d_{n}^{3}/\gamma \right)}^{1/2}$. The viscosity was non-dimensionalized as $\mu {}^{\prime }=\mu /{\left(\gamma \rho d_{n}\right)}^{1/2}$. The nozzle-to-substrate distance was non-dimensionalized as $H{}^{\prime }=H/d_{n}$. The applied voltage was non-dimensionalized according to Equation (11). Each variable was varied over five levels: factorial points ±2, ±1, and center point 0. The different values for different levels of conductivities, viscosities, and nozzle-to-substrate distances are listed in Table 1. The applied voltage changed with nozzle-to-substrate distance according to Equation (11). Its values for different nozzle-to-substrate distances and different levels are listed in Table 2. The dimensionless values of the different levels of the four independent parameters are listed in Table 3.

Design-Expert software was used for the analysis of the experimental data. The experiments were designed by the central composite design (CCD) method. The experimental strategy and experimental results are listed in Table A2. The center point experiment was repeated six times. There was a total of 30 experiments for the regression analysis. Polynomials were used to fit the data. The higher the order of the polynomial, the better the polynomial fits to the experimental results. In this case, a third-order polynomial was used to fit the experimental data. It can be written as follows:

(12) $$\begin{array}{l} Y=\beta_{0}+\sum_{i=1}^{k}\beta_{i}x_{i}+\sum_{i=1}^{k}\beta_{ii}x_{i}^{2}+\sum_{i=1}^{k}\beta_{iii}x_{i}^{3}+\sum_{i=1}^{k-1}\sum_{j=i+1}^{k}\beta_{ij}x_{i}x_{j}+\\ \sum_{i=1}^{k-2}\sum_{j=i+1}^{k-1}\sum_{m=j+1}^{k}\beta_{ijm}x_{i}x_{j}x_{m}+\sum_{i=1}^{k-1}\sum_{j=i+1}^{k}\beta_{iij}x_{i}^{2}x_{j} \end{array}$$

where Y is the response variable, which is the dimensionless ejection volume in our experiments. x represents the independent variable. β is the coefficient of the polynomial. The fitting result of the polynomial is

(13) $$\begin{array}{l} V{}^{\prime }=1.01485-(9.79041e-7)\cdot K{}^{\prime }+0.28325\cdot \mu {}^{\prime }-2.08555\cdot U{}^{\prime }-(1.65541e-3)\cdot H{}^{\prime }\\ -(1.23168e-6)\cdot K{}^{\prime }\cdot \mu {}^{\prime }+(1.32518e-6)\cdot K{}^{\prime }\cdot U{}^{\prime }+(9.63912e-9)\cdot K{}^{\prime }\cdot H{}^{\prime }\\ -0.26589\cdot \mu {}^{\prime }\cdot U{}^{\prime }-0.030864\cdot \mu {}^{\prime }\cdot H{}^{\prime }+(1.04634E-3)\cdot U{}^{\prime }\cdot H{}^{\prime }-(6.02452e-12)\cdot K{{}^{\prime }}^{2}\\ -(0.010957)\cdot \mu {{}^{\prime }}^{2}+1.07092\cdot U{{}^{\prime }}^{2}+0.032631\cdot \mu {}^{\prime }\cdot U{}^{\prime }\cdot H{}^{\prime }+(1.84190e-11)\cdot K{{}^{\prime }}^{2}\cdot \mu {}^{\prime } \end{array}$$

The adjusted R-square value is 0.9920, which indicates the regression model fits the experimental data very well. Analysis of variance (ANOVA) was used to check the significance of the model. The results are listed in Table 4. The F-value indicates the significance of the model and each term. A large F-value means that the model captures most of the variance in the dimensionless ejection volume ($V{}^{\prime }$). The *p*-value (Prob > F) means the possibility that a “Model F-value” or “Term F-value” this large could occur due to noise. When the *p*-value (Prob > F) is less than 0.05, the terms are significant. The terms with *p*-value (Prob > F) larger than 0.1 are not significant and ignored in the regression model. The F-value of the model is 241.34 and the *p*-value of the model is less than 0.0001. So, the model is significant. The “Lack of fit” F-value is 2.06 and the “Lack of fit” *p*-value is 0.2212. The lack of fit is not significant. That means the model fits the results well. If a second-order polynomial is used, the “Lack of fit” F-value is 11.70 and the “Lack of fit” *p*-value is 0.0067. The lack of fit is significant, which means the second-order polynomial cannot fit the experimental data well. So, the third-order polynomial is the most simplified model that can fit the experimental data well. Sixteen terms are included in the model. Thirteen model terms are significant, which are $K{}^{\prime }$, $\mu {}^{\prime }$, $U{}^{\prime }$, $H{}^{\prime }$, $K{}^{\prime }\mu {}^{\prime }$, $K{}^{\prime }U{}^{\prime }$, $K{}^{\prime }H{}^{\prime }$, $\mu {}^{\prime }U{}^{\prime }$, $U{}^{\prime }H{}^{\prime }$, $K{{}^{\prime }}^{2}$, $\mu {{}^{\prime }}^{2}$, $U{{}^{\prime }}^{2}$, and $K{{}^{\prime }}^{2}\mu {}^{\prime }$. From the ANOVA results, the linear terms for all four parameters are significant. For the conductivity, the viscosity, and the applied voltage, the square terms are also significant. Some of the cross-terms are also significant. The cross-terms contain all four parameters that influence the ejection volume, which means the increase/decrease rate of the ejection volume with each parameter is influenced by the values of the other parameters.

**Figure 7:**
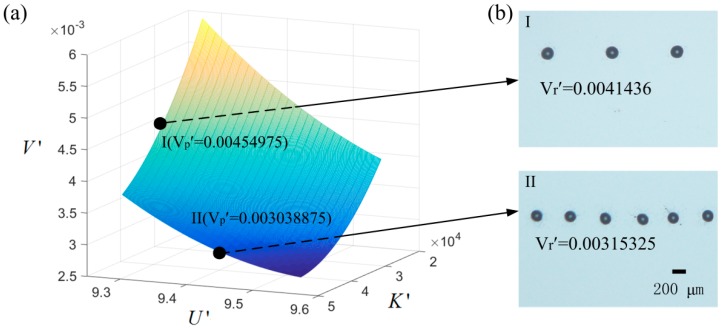
(**a**) A surface of the predicted dimensionless ejection volume of two points in the operation field: I ($K{}^{\prime }$ = 3.857 × 10^4^, $U{}^{\prime }$ = 0.9228) and II ($K{}^{\prime }$ = 5.142 × 10^4^, $U{}^{\prime }$ = 0.9358). $\mu {}^{\prime }$ = 0.4224 and $H{}^{\prime }$ = 5.769 for the two cases. (**b**) The printed droplets on the substrate at the conditions of I and II. $V_{p}{}^{\prime }$ represents the predicted dimensionless ejection volume from the regression model. $V_{r}{}^{\prime }$ represents the real dimensionless ejection volume of the experiments.

In order to verify the validity of the model, two more points were run, as shown in Figure 7. The dimensionless ejection volumes of the two points are 0.004143625 ($K{}^{\prime }$ = 3.857 × 10^4^, $\mu {}^{\prime }$ = 0.4224, $U{}^{\prime }$ = 0.9228, $H{}^{\prime }$ = 5.769) and 0.00315325 ($K{}^{\prime }$ = 5.142 × 10^4^, $\mu {}^{\prime }$ = 0.4224, $U{}^{\prime }$ = 0.9358,$H{}^{\prime }$ = 5.769) in the experiments. The images of the printed dots are shown in Figure 7. The predicted dimensionless ejection volumes of the two points by the model are 0.00459139 and 0.003081479, respectively. By comparison, the error between the experimental value and the prediction value is less than 10%. So, the model gives a good prediction of the experimental results. The model can be used to design the printing parameters to get the right ejection volume.

## 5. Conclusions

In this study, the influence of viscosity, conductivity, applied voltage, and nozzle-to-substrate distance on the ejection volume in e-jet printing was analyzed through experiments. The ejection volume increases as the viscosity of the liquid increases. The ejection volume decreases with the increase of the conductivity of the liquid. Because the electric shear stress is weaker for high-conductivity liquid, less liquid is pulled out for high-conductivity liquid. The relation of the ejection volume and conductivity is $V\sim K^{-0.6}$, which is different from the previous theory, which concluded that $V\sim K^{-1}$. The ejection volume decreases with the applied voltage. That is caused by the decrease of the Taylor cone volume as the applied voltage increases. The ejection volume decreases with the nozzle-to-substrate distance. The average electric field is kept unchanged while changing the nozzle-to-substrate distance by changing the applied voltage according to the electric field model of a semi-infinite wire perpendicular to an infinite large planar counter electrode. Finally, regression analysis was used to get a model describing the relation between the ejection volume and the four parameters. The central composite design (CCD) method was used to design the experiments for the regression analysis. Analysis of variance (ANOVA) showed that the model is significant. Sixteen terms are included in the model. Thirteen model terms are significant, including four linear terms ($K{}^{\prime }$, $\mu {}^{\prime }$, $U{}^{\prime }$, $H{}^{\prime }$), three square terms ($K{{}^{\prime }}^{2}$, $\mu {{}^{\prime }}^{2}$, $U{{}^{\prime }}^{2}$), and six cross-terms ($K{}^{\prime }\mu {}^{\prime }$, $K{}^{\prime }U{}^{\prime }$, $K{}^{\prime }H{}^{\prime }$, $\mu {}^{\prime }U{}^{\prime }$, $U{}^{\prime }H{}^{\prime }$, $K{{}^{\prime }}^{2}\mu {}^{\prime }$). The cross-terms contain all four parameters that influence the ejection volume, which means that the increase/decrease rate of the ejection volume with each parameter is influenced by the values of the other parameters. Two more experiments were done, and the prediction value of the regression model accorded well with the experiments. The model can be used to choose the appropriate parameters to get the right ejection volume.

## Figures and Tables

**Table 1:** The different values for different levels of conductivities (µS/cm), viscosities (mPa·s), and nozzle-to-substrate distances (mm).

Levels				
−2	−1	0	1	2
2	4	6	8	10
20	40	60	80	100
1	1.25	1.5	1.75	2

**Table 2:** The applied voltage values for different nozzle-to-substrate distances and different levels.

*H* (mm)	Levels				
	−2	−1	0	1	2
1	1868.9 V	1895.7 V	1922.5 V	1949.3 V	1976.2 V
1.25	1990.6 V	2019.1 V	2047.7 V	2076.3 V	2104.9 V
1.5	2090 V	2120 V	2150 V	2180 V	2210 V
1.75	2174.1 V	2205.3 V	2236.5 V	2267.7 V	2298.9 V
2	2246.9 V	2279.2 V	2311.4 V	2343.7 V	2375.9 V

**Table 3:** Dimensionless parameter values for different levels.

Parameters	Levels				
	−2	−1	0	1	2
$K{}^{\prime }$	1.286 × 10^4^	2.571 × 10^4^	3.857 × 10^4^	5.142 × 10^4^	6.428 × 10^4^
$\mu {}^{\prime }$	0.1408	0.2816	0.4224	0.5632	0.7040
$U{}^{\prime }$	0.9097	0.9228	0.9358	0.9489	0.9619
$H{}^{\prime }$	3.846	4.808	5.769	6.731	7.692

**Table 4:** Analysis of variance (ANOVA).

Source	Sum of Squares	df	Mean Square	F Value	*p-*Value Prob > F	Significance
Model	5.88 × 10^−5^	15	3.92 × 10^−6^	241.34	<0.0001	significant
$K{}^{\prime }-K{}^{\prime }$	1.84 × 10^−5^	1	1.84 × 10^−5^	1135.12	<0.0001	
$\mu {}^{\prime }-\mu {}^{\prime }$	1.59 × 10^−6^	1	1.59 × 10^−6^	98.22	<0.0001	
$U{}^{\prime }-U{}^{\prime }$	1.32 × 10^−5^	1	1.32 × 10^−5^	814.39	<0.0001	
$H{}^{\prime }-H{}^{\prime }$	4.36 × 10^−6^	1	4.36 × 10^−6^	268.34	<0.0001	
$K{}^{\prime }\mu {}^{\prime }$	1.87 × 10^−6^	1	1.87 × 10^−6^	115.34	<0.0001	
$K{}^{\prime }U{}^{\prime }$	7.92 × 10^−7^	1	7.92 × 10^−7^	48.78	<0.0001	
$K{}^{\prime }H{}^{\prime }$	2.27 × 10^−7^	1	2.27 × 10^−7^	13.99	0.0022	
$\mu {}^{\prime }U{}^{\prime }$	3.26 × 10^−7^	1	3.26 × 10^−7^	20.08	0.0005	
$\mu {}^{\prime }H{}^{\prime }$	3.15 × 10^−8^	1	3.15 × 10^−8^	1.94	0.1850	
$U{}^{\prime }H{}^{\prime }$	5.55 × 10^−7^	1	5.55 × 10^−7^	34.17	<0.0001	
$K{{}^{\prime }}^{2}$	2.36 × 10^−6^	1	2.36 × 10^−6^	145.13	<0.0001	
$\mu {{}^{\prime }}^{2}$	1.32 × 10^−6^	1	1.32 × 10^−6^	81.39	<0.0001	
$U{{}^{\prime }}^{2}$	9.34 × 10^−7^	1	9.34 × 10^−7^	57.53	<0.0001	
$\mu {}^{\prime }U{}^{\prime }H{}^{\prime }$	5.32 × 10^−8^	1	5.32 × 10^−8^	3.28	0.0916	
$K{{}^{\prime }}^{2}\mu {}^{\prime }$	9.79 × 10^−7^	1	9.79 × 10^−7^	60.35	<0.0001	
Residual	2.27 × 10^−7^	14	1.62 × 10^−8^			
Lack of Fit	1.79 × 10^−7^	9	1.99 × 10^−8^	2.06	0.2212	not significant
Pure Error	4.84 × 10^−8^	5	9.67 × 10^−9^			
Cor Total	5.90 × 10^−5^	29				

## Abbreviations

**The Nomenclature of the Variables**	
ρ	The density of the liquid
γ	The surface tension of the liquid
$d_{n}$	The nozzle outer diameter
f	The pulsation frequency of the jet ejection under constant applied voltage
$\varepsilon_{0}$	The dielectric constant of air
$\varepsilon_{r}$	The relative dielectric constant of the liquid
μ	The viscosity of the liquid
U	The applied voltage
H	The nozzle-to-substrate distance
V	The ejection volume
$Q_{e}$	The equivalent flow rate on the jet
$E_{n}$	The normal electric field on the meniscus
$Q_{s}$	The supplied flow rate
K	The conductivity of the liquid
$\tau_{s}$	The electric shear stress
$E_{s}$	The electric shear field
σ	The surface charge density
$\sigma_{e}$	The equilibrium surface charge density
I	The current emitted by the jet
$r_{c}$	The diameter of some point at the cone surface
$d_{j}$	The jet diameter
$t_{e}=\varepsilon_{r}\varepsilon_{0}/K$	The charge relaxation time
$t_{c}={\left(\rho d_{n}^{3}/\gamma \right)}^{1/2}$	The capillary time
$L_{n}$	The length of the nozzle
$U{}^{\prime }$	The dimensionless applied voltage
$V{}^{\prime }$	The dimensionless ejection volume
$K{}^{\prime }$	The dimensionless conductivity
$H{}^{\prime }$	The dimensionless nozzle-to-substrate distance

## Appendix A

**Table A1:** The compositions and properties of the liquids used in the experiments.

Composition (Glycerine (mL) + Water (mL) + NaCl Solution (mL))	*µ* (mPa·s)	*K* (µS/cm)	*ρ* $\;(\;kg/m^{3})$	*γ* (mN/m)	Composition (Glycerine (mL) + Water (mL) + NaCl Solution (mL))	*µ* (mPa·s)	*K* (µS/cm)	*ρ* $\;(\;kg/m^{3})$	*γ* (mN/m)
10 + 3.95 + 0.05	40	2	1194.3	65	10 + 2.41 + 0.09	80	2	1219.5	64.2
10 + 3.91 + 0.09	40	4	1194.3	65	10 + 2.32 + 0.18	80	4	1219.5	64.2
10 + 3.86 + 0.14	40	6	1194.3	65	10 + 2.22 + 0.28	80	6	1219.5	64.2
10 + 3.82 + 0.18	40	8	1194.3	65	10 + 2.14 + 0.36	80	8	1219.5	64.2
10 + 3.78 + 0.22	40	10	1194.3	65	10 + 2.06 + 0.44	80	10	1219.5	64.2
10 + 2.92 + 0.08	60	2	1208.4	64.5	10 + 4.42 + 0.08	30	4	1184.8	65.2
10 + 2.85 + 0.15	60	4	1208.4	64.5	10 + 4.38 + 0.12	30	6	1184.8	65.2
10 + 2.79 + 0.21	60	6	1208.4	64.5	10 + 4.34 + 0.16	30	8	1184.8	65.2
10 + 2.72 + 0.28	60	8	1208.4	64.5	10 + 4.9 + 0.1	20	6	1172.2	65.7
10 + 2.65 + 0.35	60	10	1208.4	64.5	10 + 2.0 + 0.3	100	6	1219.6	64.1

The NaCl aqueous solution was prepared by 0.5 g NaCl dissolved into 80 mL deionized water.

**Table A2:** The experiments and the experimental results.

*K* (µS/cm)		*µ* (mPa·s)	*H* (mm)	*U* (V)	*V* (pL)	*K* (µS/cm)		*µ* (mPa·s)	*H* (mm)	*U* (V)	*V* (pL)
1	4 (−1)	40 (−1)	1.25 (−1)	2019.1 (−1)	103.31	16	8 (1)	80 (1)	1.75 (1)	2267.7 (1)	58.86
2	8 (1)	40 (-1)	1.25 (−1)	2019.1 (−1)	42.40	17	2 (-2)	60 (0)	1.5 (0)	2150 (0)	115.23
3	4 (−1)	80 (1)	1.25 (−1)	2019.1 (−1)	126.72	18	10 (2)	60 (0)	1.5 (0)	2150 (0)	55.61
4	8 (1)	80 (1)	1.25 (−1)	2019.1 (−1)	97.71	19	6 (0)	20 (-2)	1.5 (0)	2150 (0)	34.06
5	4 (−1)	40 (−1)	1.75 (1)	2205.3 (−1)	66.68	20	6 (0)	100 (2)	1.5 (0)	2150 (0)	65.45
6	8 (1)	40 (−1)	1.75 (1)	2205.3 (−1)	28.89	21	6 (0)	60 (0)	1 (-2)	1922.5 (0)	105.00
7	4 (−1)	80 (1)	1.75 (1)	2205.3 (−1)	80.17	22	6 (0)	60 (0)	2 (2)	2311.4 (0)	50.74
8	8 (1)	80 (1)	1.75 (1)	2205.3 (−1)	65.95	23	6 (0)	60 (0)	1.5 (0)	2090 (−2)	76.87
9	4 (−1)	40 (−1)	1.25 (−1)	2076.3 (1)	75.73	24	6 (0)	60 (0)	1.5 (0)	2210 (2)	50.48
10	8 (1)	40 (−1)	1.25 (−1)	2076.3 (1)	32.34	25	6 (0)	60 (0)	1.5 (0)	2150 (0)	62.35
11	4 (−1)	80 (1)	1.25 (−1)	2076.3 (1)	97.79	26	6 (0)	60 (0)	1.5 (0)	2150 (0)	65.48
12	8 (1)	80 (1)	1.25 (−1)	2076.3 (1)	74.65	27	6 (0)	60 (0)	1.5 (0)	2150 (0)	63.86
13	4 (−1)	40 (−1)	1.75 (1)	2267.7 (1)	53.59	28	6 (0)	60 (0)	1.5 (0)	2150 (0)	65.69
14	8 (1)	40 (−1)	1.75 (1)	2267.7 (1)	22.42	29	6 (0)	60 (0)	1.5 (0)	2150 (0)	64.26
15	4 (−1)	80 (1)	1.75 (1)	2267.7 (1)	69.56	30	6 (0)	60 (0)	1.5 (0)	2150 (0)	67.35

The number outside the bracket represents the real value of the parameter, the number in the bracket represents the level of the parameter.

## References

[1] Shigeta K., He Y., Sutanto E., Kang S., Le P., Nuzzo R.G., Alleyne A.G., Ferreira P.M., Lu Y., Rogers J.A. (2012). Functional protein microarrays by electrohydrodynamic jet printing. Anal. Chem..

[2] Park J.U., Hardy M., Kang S.J., Barton K., Adair K., Mukhopadhyah D.K., Lee C.Y., Strano M.S., Alleyne A.G., Georgiadis J.G. (2007). High-resolution electrohydrodynamic jet printing. Nat. Mater..

[3] Onses M.S., Song C., Williamson L., Sutanto E., Ferreira P.M., Alleyne A.G., Nealey P.F., Ahn H., Rogers J.A. (2013). Hierarchical patterns of three-dimensional block-copolymer films formed by electrohydrodynamic jet printing and self-assembly. Nat. Nanotechnol..

[4] Mao M., He J., Li X., Zhang B., Lei Q., Liu Y., Li D. (2017). The emerging frontiers and applications of high-resolution 3D printing. Micromachines.

[5] Huang Y.A., Ding Y., Bian J., Su Y., Zhou J., Duan Y., Yin Z. (2017). Hyper-stretchable self-powered sensors based on electrohydrodynamically printed, self-similar piezoelectric nano/microfibers. Nano Energy.

[6] Bu N., Huang Y., Duan Y., Ding Y., Yin Z. (2015). Near-field behavior of electrified jet under moving substrate constrains. AIP Adv..

[7] Lei T., Lu X., Yang F. (2014). Fabrication of various micro/nano structures by modified near-field electrospinning. AIP Adv..

[8] Ye D., Ding Y., Duan Y., Su J., Yin Z., Huang Y. (2018). Large-scale direct-writing of aligned nanofibers for flexible electronics. Small.

[9] Schneider J., Rohner P., Thureja D., Schmid M., Galliker P., Poulikakos D. (2016). Electrohydrodynamic nanodrip printing of high aspect ratio metal grid transparent electrodes. Adv. Funct. Mater..

[10] Park J.U., Lee J.H., Paik U., Rogers J.A. (2008). Nanoscale patterns of oligonucleotides formed by electrohydrodynamic jet printing with applications in biosensing and nanomaterials assembly. Nano Lett..

[11] Kim B.H., Onses M.S., Lim J.B., Nam S., Oh N., Kim H., Yu K.J., Lee J.W., Kim J.H., Kang S.K. (2015). High-resolution patterns of quantum dots formed by electrohydrodynamic jet printing for light-emitting diodes. Nano Lett..

[12] Galliker P., Schneider J., Eghlidi H., Kress S., Sandoghdar V., Poulikakos D. (2012). Direct printing of nanostructures by electrostatic autofocussing of ink nanodroplets. Nat. Commun..

[13] Han Y., Wei C., Dong J. (2014). Super-resolution electrohydrodynamic (EHD) 3D printing of micro-structures using phase-change inks. Manuf. Lett..

[14] Wang D., Zha W., Feng L., Qian M., Liu X., Yang N., Xu Z., Zhao X., Liang J., Ren T. (2016). Electrohydrodynamic jet printing and a preliminary electrochemistry test of graphene micro-scale electrodes. J. Micromech. Microeng..

[15] Song C.H., Back S.Y., Yu S.L., Lee H.J., Kim B.S., Yang N.Y., Jeong S.H., Ahn H. (2012). Direct-patterning of porphyrin dot arrays and lines using electrohydrodynamic jet printing. J. Nanosci. Nanotechnol..

[16] Lim S., Park S.H., An T.K., Lee H.S., Kim S.H. (2016). Electrohydrodynamic printing of poly(3,4-ethylenedioxythiophene):poly(4-styrenesulfonate) electrodes with ratio-optimized surfactant. RSC Adv..

[17] Xu L., Wang X., Lei T., Sun D., Lin L. (2011). Electrohydrodynamic deposition of polymeric droplets under low-frequency pulsation. Langmuir.

[18] Deng W., Gomez A. (2012). Full transient response of Taylor cones to a step change in electric field. Microfluid. Nanofluid..

[19] Pan Y., Huang Y., Guo L., Ding Y., Yin Z. (2015). Addressable multi-nozzle electrohydrodynamic jet printing with high consistency by multi-level voltage method. AIP Adv..

[20] Lee M.W., Kim N.Y., Yoon S.S. (2013). On pinchoff behavior of electrified droplets. J. Aerosol. Sci..

[21] Juraschek R., Röllgen F.W. (1998). Pulsation phenomena during electrospray ionization. Int. J. Mass Spectrom..

[22] Poon H.F. (2002). Electrohydrodynamic Printing. Ph.D. Thesis.

[23] An S., Lee M.W., Kim N.Y., Lee C.M., Al-Deyab S.S., James S.C., Yoon S.S. (2014). Effect of viscosity, electrical conductivity, and surface tension on direct-current-pulsed drop-on-demand electrohydrodynamic printing frequency. App. Phys. Lett..

[24] De La Mora J.F. (1996). On the outcome of the coulombic fission of a charged isolated drop. J. Colloid Interf. Sci..

[25] Chen C.H., Saville D.A., Aksay I.A. (2006). Scaling laws for pulsed electrohydrodynamic drop formation. Appl. Phys. Lett..

[26] Yuan X., Ba Z., Xiong Z. (2015). Fine droplet generation using tunable electrohydrodynamic pulsation. J. Micromech. Microeng..

[27] Yuan X., Xiong Z. (2018). High frequency pulsed electrohydrodynamic printing with controllable fine droplets. J. Micromech. Microeng..

[28] Rahman K., Ali K., Muhammad N.M., Hyun M., Choi K. (2013). Fine resolution drop-on-demand electrohydrodynamic patterning of conductive silver tracks on glass substrate. Appl. Phys. A.

[29] Ball A.K., Das R., Roy S.S., Kisku D.R., Murmu N.C. Prediction of droplet diameter in E-jet printing using statistical method. Proceedings of the International Conference on Sustainable Manufacturing, Automation and Robotics Technologies (IC-SMART).

[30] Das R., Ball A.K., Roy S.S. (2018). Parametric Optimization of E-Jet Based Micro Manufacturing System through Hybrid Taguchi Methodology. Mater. Today Proc..

[31] Park J., Kim B., Kim S.Y., Hwang J. (2014). Prediction of drop-on-demand (DOD) pattern size in pulse voltage-applied electrohydrodynamic (EHD) jet printing of Ag colloid ink. Appl. Phys. A.

[32] Bober D.B., Chen C.H. (2011). Pulsating electrohydrodynamic cone-jets: From choked jet to oscillating cone. J. Fluid Mech..

[33] Collins R.T., Jones J.J., Harris M.T., Basaran O.A. (2008). Electrohydrodynamic tip streaming and emission of charged drops from liquid cones. Nat. Phys..

[34] Choi H.K., Park J.U., Park O.O., Ferreira P.M., Georgiadis J.G., Rogers J.A. (2008). Scaling laws for jet pulsations associated with high-resolution electrohydrodynamic printing. App. Phys. Lett..

[35] Hayati I., Bailey A.I., Tadros T.F. (1987). Investigations into the mechanisms of electrohydrodynamic spraying of liquids: I. Effect of electric field and the environment on pendant drops and factors affecting the formation of stable jets and atomization. J. Colloid Interface Sci..

